# An ecological study of sand flies (Diptera: Psychodidae) in the vicinity of Lençóis Maranhenses National Park, Maranhão, Brazil

**DOI:** 10.1186/s13071-015-1045-5

**Published:** 2015-08-28

**Authors:** Adalberto Alves Pereira Filho, Maria da Conceição Abreu Bandeira, Raquel Silva Fonteles, Jorge Luiz Pinto Moraes, Camila Ragonezi Gomes Lopes, Maria Norma Melo, José Manuel Macário Rebêlo

**Affiliations:** Departamento de Parasitologia, Instituto de Ciências Biológicas, Universidade Federal de Minas Gerais, 31270-901 Belo Horizonte, MG Brazil; Departamento de Biologia, Centro de Ciências Biológicas e da Saúde, Universidade Federal do Maranhão, 65080-805 São Luís, MA Brazil; Departamento de Cartografia, Instituto de Geociências, Universidade Federal de Minas Gerais, 31270-901 Belo Horizonte, MG Brazil

**Keywords:** Tegumentary leishmaniasis, Sand flies, *Lutzomyia*, Abundance

## Abstract

**Background:**

The Lençóis Maranhenses National Park, located in Maranhão, Brazil, is a region of exceptional beauty and a popular tourist destination. The adjoining area has suffered from the impact of human activity and, consequently, has experienced outbreaks of leishmaniasis. This study aimed to evaluate the composition, abundance, species richness and seasonal distribution of sand flies in the region and to determine the constancy of the insect population.

**Methods:**

The survey was conducted at three sites located in the municipalities of Barreirinhas and Santo Amaro between September 2012 and August 2013. Sampling was performed monthly using automatic light traps installed 1.5 m above the soil adjacent to 13 randomly selected rural dwellings. At each site, one trap was placed in the peridomicile near to animal enclosures and another (extradomicile) at 500 m from the peridomicile.

**Results:**

A total of 4,474 individual sand flies were collected over the year with the highest abundance recorded during the rainy season (December to June). Nine species were collected: *L. whitmani*, *L. longipalpis*, *L. lenti*, *L. sordellii*, *L. evandroi*, *L. flaviscutellata*, *L. wellcomei*, *L. termitophila* and *L. intermedia*. Although peridomiciliary and extradomiciliary environments presented similar species richness, the Shannon diversity index was significantly lower in the former (*H’* = 2.4) compared with the latter (*H’* = 4.98). *Lutzomyia whitmani* and *L. longipalpis* were the most abundant species and were classified as constant (constancy index, CI = 100 %) along with *L. lenti* (CI = 58.3), *L. evandroi* (CI = 58.3) and *L. sordellii* (CI = 66.7). The remaining four species presented CI values between 25 and 50 % and were considered accessory.

**Conclusions:**

The present results confirm the present of *L. whitmani* and *L. longipalpis* in the peridomicile of houses in Lençóis National Park. The abundance of these species could explain, respectively, the endemicity of cutaneous leishmaniasis and sporadic cases of visceral leishmaniasis in the study area. However, in the case of cutaneous leishmaniasis, the presence of other sand fly vectors (in addition to *L. whitmani*) cannot be neglected. Finally, this study emphasizes the need for a more effective and permanent supervision to control the expansion of these vectors and leishmaniasis outbreaks.

## Background

Leishmaniasis comprises a group of parasitic diseases caused by protozoa of the genus *Leishmania* (Kinetoplastida: Trypanosomatidae). The most common forms of the disease are tegumentary leishmaniasis (TL), with around one million cases recorded globally in the last five years, and visceral leishmaniasis (VL), of which it is estimated that 200,000 to 400,000 new cases occur worldwide each year. However, more than 90 % of notified cases occur in half of the six countries including Brazil, some regions of which exhibit high endemicity for the disease [1].

The principal mechanism of transmission of *Leishmania* spp. to mammals, including humans, is through the bite of *Leishmania*-infected female sand fly (Diptera: Psychodidae: Phlebotominae) of the genera *Lutzomyia* in the New World and *Phlebotomus* in Old World [2]. In Brazil, epidemiological surveys carried out in rural and peri-urban areas of the northern state of Maranhão (MA) with recorded cases of TL and VL revealed the presence of at least 91 species of sand flies, of which 87 belonged to the genus *Lutzomyia* and 4 were of the genus *Brumptomyia* [3]. Species diversity was very high within primary forest environments but decreased somewhat in altered primary and secondary forests [4, 5]. Moreover, sand flies were found infesting the man-made environment, particularly dwellings associated with domestic animals, where the possibility of feeding on human blood was considerably enhanced [6].

One of the main regions of *Leishmania* transmission in Maranhão encompasses the important conservation zone designated Lençóis Maranhenses National Park (2°19 '- 2°45' S; 42°44 '- 43°29' W). This protected area, which comprises 155,000 ha with a perimeter of 270 km, is characterized by large dunes of white sand interspersed with lagoons and represents, a key tourist destination in the state. While the park itself extends through the municipalities of Barreirinhas, Santo Amaro and Primeira Cruz, the main point of entry is via Barreirinhas [7]. This municipality and its adjacent areas have, therefore, received various tourism-related investments over the years with the emergence of numerous developments and the expansion of municipal headquarters. The accompanying growth in the number of inhabitants, which has risen from 29,640 in 1991 to 43,000 in 2010 [8], and the increase in tourist activity have exerted significant cultural, economical and environmental impacts on the area with consequential public health problems, including outbreaks of leishmaniasis [9]. During the period 2000 to 2008 some 737 cases of TL were notified in the town of Barreirinhas alone, with high coefficients of detection per 100,000 inhabitants in 2000 (308.2), 2001 (310.9), 2002 (338.2) and 2005 (313.6). Furthermore, between 2009 and 2014 the number of cases remained high with 453 notified occurrences [10], placing this town in a prominent position in the state scenario of leishmaniasis [9].

Preliminary studies carried out in Barreirinhas and the surrounding villages revealed that the main sand flies present were the recognized *Leishmania* vectors, *Lutzomyia whitmani*, *L. longipalpis* and *L. flaviscutellata* [11]. However, no studies concerning the structure of the insect communities in the area or the epidemiology of the disease have been performed so far. The aims of the present investigation were to evaluate the composition, abundance, species richness and seasonal distribution of sand flies in the Lençóis Maranhenses National Park, and to determine the constancy index of the insect populations.

## Methods

The collection of sand flies was authorized by Instituto Chico Mendes de Conservação da Biodiversidade (ICMBio) register: Sisbio/ICMbio 46319–1. The objectives of the project were explained to the owners of the farms where the light traps would be exposed, and they were then invited to take part in the project and sign an informed consent.

### Study area

Surveys were carried out in two municipalities of Maranhão State. Barreirinhas (2°45'S; 42°5'W), located 266 km from the state capital São Luis, encompasses an area of 3,111 km^2^ and 67 % of its 43,000 inhabitants reside in rural districts [8]. Santo Amaro (2°30′S; 43°15′W), located 243 km from the state capital, encompasses an area of 1,601 km^2^ and 73 % of its 13,820 inhabitants reside in rural districts [8]. The study area has a semi-humid tropical climate with a mean annual rainfall of 1,900 mm, the major portion of which (96 %) occurs during the rainy season from December to June while the remaining 4 % falls sporadically in the dry season from July to November.

### Insect sampling

**Fig. 1:**
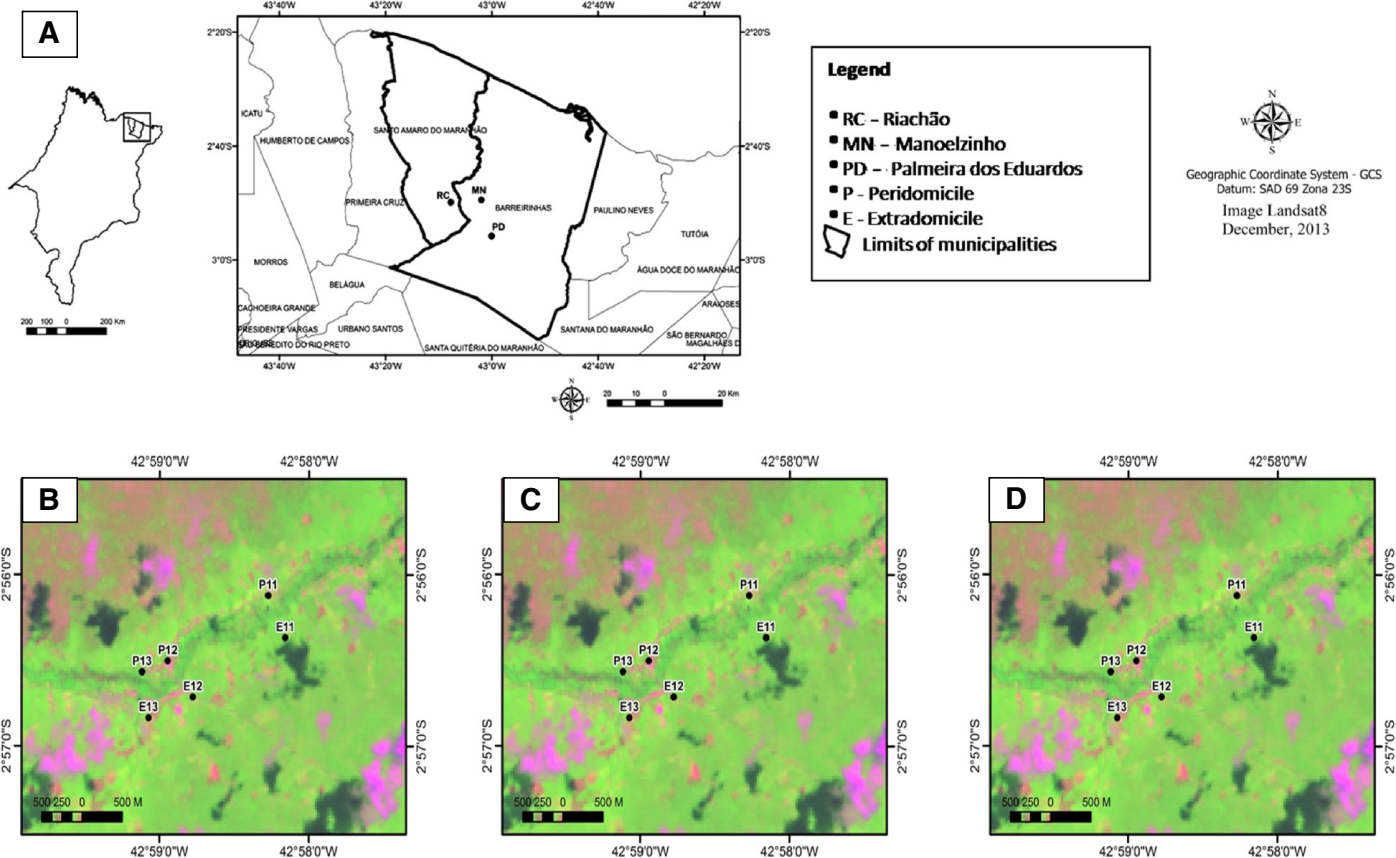
Localization of the Brazilian State of Maranhão and the municipalities in which the Lençóis Maranhenses National Park is situated **a**. Spatial arrangement of the sampling sites located at Riachão in the municipality of Santo Amaro **b**, and at Manoelzinho (**c**) and Palmeira dos Eduardos (**d**) located in the municipality of Barreirinhas

From September 2012 to August 2013, 26 automatic HP light traps [12] were installed at 1.5 m above the soil in the surroundings of 13 randomly selected rural dwellings named Manoelzinho (*n* = 5), Palmeira dos Eduardos (*n* = 3), both located in Barreirinhas municipality, and Riachão (*n* = 5), located in Santo Amaro municipality. Each dwelling received two traps, one placed within the peridomicile near to animal enclosures (chicken pens, pigsties or cowsheds) wherever possible, and the other located 500 m from the peridomicile in the extradomiciliary environment [the sandy coastal environment (forest)] (Fig. 1). Each trap was operated for 12 h uninterruptedly, one night per month, (from 18:00 to 06:00 h) on new moon nights, such that the effort to capture sand flies totaled 3, 744 h (i.e. 26 traps × 12 h × 12 months). The captured insects were placed in 1.5 ml plastic tubes, killed by freezing at −20 °C and transported to the Laboratory of Entomology and Vectors of the Pathology Department at the Universidade Federal do Maranhão. Sand flies were separated from the other insects and treated with a solution containing potassium hydroxide, acetic acid and lactophenol in distilled water. After clarification, specimens were placed in Berlese’s fluid and mounted individually between slides and cover slips. Sand flies were examined under the optical microscope and identified at the species level according to the dichotomous key provided by Young and Duncan [13].

### Data analysis

Data were organized in spreadsheets using Microsoft Excel 2007 and descriptive statistical analyses was performed with the aid of Statistica software version 7.0 (StatSoft South America, São Caetano do Sul, São Paulo, Brazil). The diversity of species was calculated according to the Shannon diversity index (*H’*) using PAST software version 2.04 [14]. The constancy index (CI) was employed to classify species as constant (CI > 50 %), accessory (25 % ≤ CI ≥ 50 %) or accidental (CI < 25 %) [15]. Data relating to rainfall, temperature and humidity were provided by the Núcleo Geo-Ambiental of the Universidade Estadual do Maranhão, and correlations between sand fly abundance and climatic parameters were tested by means of Spearman's rank correlation coefficient (*rs*).

## Results

### Species richness and diversity

Nine species of *Lutzomyia* were identified among the 4,474 sand fly specimens collected (Table 1). Although both peridomiciliary and extradomiciliary environments presented similar values for species richness, the Shannon diversity index was considerably lower in the former settings (*H’* = 2.4) than in the latter (*H’* = 4.98).

**Table 1:** Distribution according to sex and area of phlebotominae sand fly collected at three locations in the municipalities of Barreirinhas and Santo Amaro, MA, Brazil

Species	Sex *n* (%)		Area *n* (%)		Total (%)
	Male	Female	Peridomicile	Extradomicile	
*L. whitmani* (Antunes & Coutinho, 1939)	1,242 (51.03)	854 (41.86)	1,748 (50.49)	348 (34.39)	2,096 (46.85)
*L. longipalpis* (Lutz &Neiva, 1912)	987 (40.55)	948 (46.47)	1,589 (45.90)	346 (34.19)	1,935 (43.25)
*L. lenti* (Mangabeira, 1938)	100 (4.11)	64 (3.14)	56 (1.62)	108 (10.67)	164 (3.67)
*L. sordellii* (Shannon & Del Ponte, 1927)	60 (2.47)	45 (2.21)	20 (0.58)	85 (8.40)	105 (2.35)
*L. flaviscutellata* (Mangabeira Fo, 1942)	18 (0.74)	55 (2.70)	9 (0.26)	64 (6.32)	73 (1.63)
*L. evandroi* (Costa Lima & Antunes, 1936)	11 (0.45)	31 (1.52)	28 (0.81)	14 (1.38)	42 (0.94)
*L. termitophila* (Martins, Falcão & Silva, 1964)	6 (0.25)	16 (0.78)	6 (0.17)	16 (1.58)	22 (0.49)
*L. intermedia* (Lutz & Neiva, 1912)	3 (0.12)	17 (0.83)	1 (0.03)	19 (1.88)	20 (0.45)
*L. wellcomei* (Fraiha, Shaw & Lainson, 1971)	7 (0.29)	10 (0.49)	5 (0.14)	12 (1.19)	17 (0.38)
Total (%)	2,434 (54.40)	2,040 (45.60)	3,462 (77.40)	1,012 (22.60)	4,474 (100,00)

### Abundance of sand flies

The most abundant sand fly species were *L. whitmani* (46.85), *L. longipalpis* (43.25), *L. lenti* (3.67), *L. sordellii* (2.35) and *L. flaviscutellata* (1.63 %), while the remaining four species together accounted for only 2.26 % (Table 1). In general, male sand flies predominated over females (54 and 46 %, respectively), although this distribution was influenced mainly by the abundance of *L. whitmani* and *L. longipalpis* males, which contributed 51.03 and 40.55 %, respectively, of all specimens collected.

In the peridomiciliary settings, the most abundant species were *L. whitmani* (50.49) and *L. longipalpis* (45.90 %), while the remaining seven species accounted for only 3.61 % of the total number of specimens captured. In the extradomiciliary environments, the most abundant species were *L. whitmani* (34.39 %), *L. longipalpis* (34.19 %), *L. lenti* (10.67 %), *L. sordellii* (8.40 %) and *L. flaviscutellata* (6.32 %), with the remaining four species accounting for 6.03 % of the total number of specimens trapped (Table 1).

The relative abundance of species was significantly greater in peridomiciliary environments than in extradomiciliary settings (*F* = 4.95; degrees of freedom = 1; *P* < 0.01), and the total number of sand flies captured in peridomicile traps was significantly higher in comparison with extradomicile traps (^χ2^ = 723. 28; degrees of freedom = 1; *P* < 0.01).

### Constancy index and seasonal distribution of sand flies

Although sand flies were found in the study areas all year around, only five species could be considered constant, *L. whitmani, L. longipalpis* (CI = 100 %), *L. sordellii* (CI = 66.7 %), *L. lenti* and *L. evandroi* (CI = 58.3 %) (Table 2). The other four species were considered accessory since they presented CI values between 25 and 50 %.

**Table 2:** Distribution according to season and constancy index of sand fly specimens captured in the municipalities of Barreirinhas and Santo Amaro, MA, Brazil

*Lutzomyia* species	Rainy season						Dry season						Total	Constancy index
	Jan	Feb	Mar	Apr	May	Jun	Jul	Aug	Sep	Oct	Nov	Dec		
*L. longipalpis*	42	87	139	274	237	229	250	349	107	60	110	51	1935	100.0
*L. whitmani*	62	279	428	406	188	127	164	149	37	99	63	94	2096	100.0
*L. lenti*	53	39	-	-	-	-	-	9	14	12	25	12	164	58.3
*L. sordellii*	26	25	-	-	-	1	-	5	4	26	11	7	105	66.7
*L. evandroi*	10	6	2	-	-	-	1	-	4	-	15	4	42	58.3
*L. flaviscutellata*	6	-	-	-	-	3	12	23	-	-	22	7	73	50.0
*L. wellcomei*	11	-	-	-	-	-	-	-	-	-	1	5	17	25.0
*L. termitophila*	9	4	-	-	-	6	1	-	-	-	2	-	22	41.7
*L. intermedia*	10	-	-	-	-	7	-	2	-	-	-	1	20	33.3
Total	229	440	569	680	425	373	428	537	166	197	249	181	4474	-

All species were present during at least one month of both dry and rainy seasons, although the distribution was somewhat irregular. Species richness was highest in January at the start of the rainy season, and representatives of all nine species were captured in the traps during this period. Subsequently, however, the frequency of some species declined such that specimens representing just two species were trapped during April and May. Species representation was more uniformly distributed during the months of the dry season, with a minimum of five species being captured throughout the period with the exception of October when only four species were captured.

**Fig. 2:**
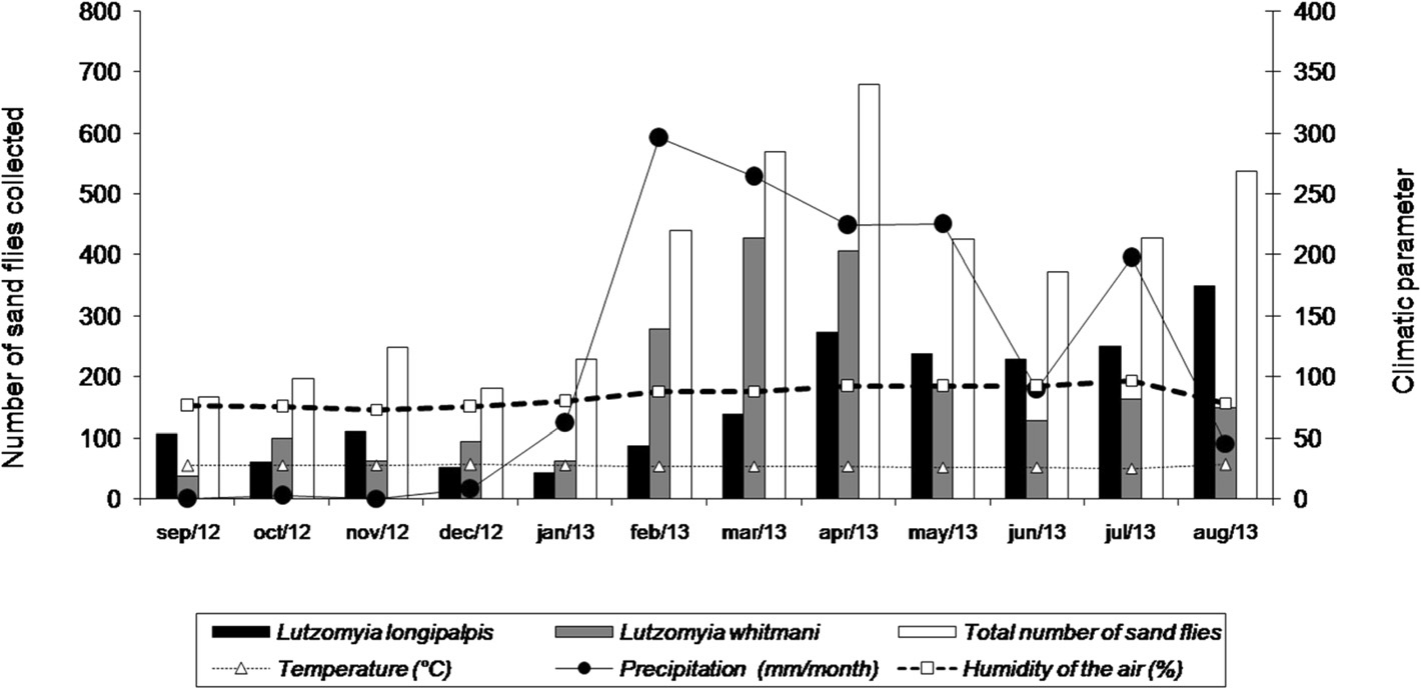
Numbers of sand flies captured at sampling sites in the municipalities of Barreirinhas and Santo Amaro, MA, Brazil, between September 2012 and August 2013 and their association with climatic factors

Regarding the pattern of species abundance, considerable numbers of sand flies were captured throughout the year, but most especially in the rainy season when the total number of specimens obtained (60.7 % of the total) was significantly higher in comparison with the dry season (^χ2^ = 103.04; degrees of freedom = 1; *P* < 0.01). The highest abundance of sand flies (680 specimens captured) was observed in the mid-rainy season month of April, whilst the lowest abundance (166 specimens captured) was recorded in the mid-dry season month of September. In any month, the abundance of sand flies was determined by the two predominant species *L. whitmani* and *L. longipalpis* (Fig. 2). Moreover, the abundance of sand flies exhibited a positive correlation with rainfall (*rs* = 0.755; *P* = 0.003) and humidity (*rs* = 0.597; *P* = 0.021) but a negative correlation with temperature (*rs* = − 0.523; *P* = 0.042).

## Discussion

Although the region comprising the Lençóis Maranhenses National Park is located on the eastern border of the tropical Amazon rainforest, its climate is semi-humid tropical. The species richness of the sand flies fauna in this area was comparable with that established for other locations in the semi-humid tropical zone typical of northeastern Brazil [16, 17], but lower than that of locations in the Amazon humid zone [5, 18, 19].

The infestation of sand flies in anthropic settings around Barreirinhas and Santo Amaro was most likely caused by deforestation and disturbance of green areas associated with urban expansion, a situation that has been observed previously in towns of southern Brazil [20]. Ramos *et al*. [21] showed that both forest cover and human population density affect sand fly diversity and abundance. These effects may be amplified when both factors are conjoined. Low forest cover can reduce sand fly numbers, but high human population density can produce environmental conditions favorable for maintaining the life cycles of several sand fly species that are adaptable to these environments [21].

Although similar sand fly species were captured in the peridomicile and extradomicile traps surveyed, the abundance of sand flies was significantly higher in the peridomiciliary settings. Martin and Rebêlo [17] reported a similar pattern of sand fly abundance in the municipality of Santa Quitéria, which is close to Barreirinhas, and suggested that the discrepancy between the settings might reflect a structural difference in the sand fly communities. If this were true, then the sand fly communities in the two settings would be characterized by species with dissimilar degrees of adaptation and diverse responses to the availability of blood sources, breeding nests and shelter conditions. According to Azevedo *et al. *[22], however, due consideration must also be given to the power of attraction of the light trap, the range of action of which is around 5 m for small insects such as sand flies [23, 24]. On this basis, it is possible that the most abundant species were sheltered close to the traps and, therefore, readily attracted, while the less common species could be accidental or occasional visitors.

Nevertheless, *L. whitmani* was the predominant species and, possibly, the best adapted to the anthropic environment since it was found in high abundance in both peridomiciliary and extradomiciliary settings of the present study and in surveys of other TL-transmission zones in Maranhão [25, 26] and in northern and south-eastern Brazil [27]. *Lutzomyia longipalpis* also favored the feeding and breeding grounds in the peridomiciliary settings of poor rural areas, thus perpetuating the VL transmission cycle among domestic animals and humans [28]. The high prevalence of these two species was expected since their presence in the area had been reported previously [3, 9], and their frequency throughout the year in modified environments has been observed in other geographical areas, as in Minas Gerais [29].

Among the other constant species identified in the present study, *L. lenti* is of particular interest because of the potential threat to human health since *L. braziliensis*-infected specimens of this species have been detected in Minas Gerais [30]. *Lutzomyia lenti* is well distributed in Brazil and is to be found in marginal areas, in the lairs of wild animals, in the shelters of domestic animals (chicken pens, pigsties and cowsheds) and in the external and internal walls of human dwellings [31]. Similarly, *L. sordellii* is reportedly present in all Brazilian regions [32], having been detected in tree trunks, rock crevices and caves, as well as inside domestic animal shelters and human domiciles [16, 33].

*Lutzomyia evandroi* is an anthropophilic sand fly that is widely distributed in Brazil and associated mainly with peridomiciliary settings [11]. The distribution of *L. evandroi* is similar to that of *L. longipalpis*, as demonstrated by research carried out in the eastern parts of Maranhão [17, 26] and in other locations [34, 35]. However, *L. evandroi* appears to be more opportunistic than *L. longipalpis* in relation to habitat, although [36] reported that the density of *L. evandroi* was higher in chicken pens than in pigsties or cowsheds. While the vectorial ability of *L. evandroi* in the transmission of human TL has not yet been demonstrated some studies indicate that this species may be implicated in the transmission of *Leishmania infantum* to dogs [37]. Furthermore, the gregarine *Ascocystis chagasi* and a non-identified trypanosomatid have been found in the gut of *Lutzomyia evandroi* during an outbreak of leishmaniasis in São Luis, the capital of Maranhão [38].

In the present study, *L. flaviscutellata* exhibited an irregular season distribution and, for this reason, it was classified as an accessory species, as mentioned previously by Barros *et al*. [39]. Although *L. flaviscutellata* was originally considered a strictly wild species, it is becoming adapted to shrubbery vegetation (secondary forests) [39], and peridomiciliary [40] and domiciliary settings. The vectorial potential of *L. flaviscutellata* in the transmission of TL has been demonstrated [41] and its presence in the environs of the Lençóis Maranhenses National Park has important epidemiological significance in the transmission of *Leishmania amazonensis* [41, 42], one of the major etiological agents of diffuse TL in Maranhão [43].

Along with *L. whitmani* and *L. intermedia, L. wellcomei* has been recognized as a vector of *L. braziliensis* in wild settings of northeastern Brazil [44]. Since *L. wellcomei* occurs almost exusively during the rainy season and maintains the typical behavior of a wild species [45], it was classified as an accessory species in the present study.

The seasonal distributions of *L. termitophila* and *L. intermedia* presented similar profiles in the present study, and both were classified as accessory species. The presence of *L. termitophila* has been reported in various Brazilian states [46] and was described as an inhabitant of termite colony [47].

With respect to *L. intermedia*, its presence at the sampling sites is of particular note since the species has not been previously detected in this area despite intensive and protracted investigations [9, 11]. However, *L. intermedia* has a widespread distribution, occurring in the Northeast, Southeast and Center-West regions of Brazil [48], and from the Atlantic coast to northern Argentina and southern Bolivia, as well as in diversified climates and altitudes [49]. Furthermore, *L. intermedia* have been reported to present a synanthropic habit [50], such also affirmed by Rangel *et al*. [51] , who described the highly anthropophilic behaviour of leishmaniasis in the state of Rio de Janeiro, Brazil. The sudden emergence of *L. intermedia* in the environs of the Lençóis Maranhenses National Park is a cause for concern and requires further detailed investigation since the species is considered a good vector for *L. braziliensis* [32]. Additionally, specimens of *L. intermedia* naturally infected with *L. braziliensis* have been found in TL endemic areas in southeastern and southern Brazil [32, 48, 52].

The abundance of sand fly specimens was significantly higher during the rainy season than in the dry season, a finding that is in accord with previous reports [53], but in conflict with some studies performed in other areas of Maranhão [17, 18]. In the present study, rainfall and humidity were positively correlated with the abundance of sand flies, whereas temperature was negatively correlated. Statistically significant correlations between climatic parameters and sand fly abundance were reported by Almeida *et al*. [54] following a study conducted in Ponta Porã, Mato Grosso do Sul, although such correlations could not be established in surveys carried out in Belo Horizonte, Minas Gerais [55] or in São Luís, MA [4]. The sites surveyed in the present study were located in an equatorial region such that the air temperature only varied between 25.0 and 28.1 °C during the year. In this case, the negative correlation between temperature and sand fly abundance could be explained by the influence of rainfall, since the largest numbers of insects were captured during periods with the highest precipitation indices, which corresponded with those presenting the lowest temperatures. However, the finding of increased numbers of sand flies following months of heavy rainfall reinforces the hypothesis that increased humidity resulting from intense precipitation promotes the emergence of winged forms of sand flies [55].

## Conclusions

This study confirms the presence of *L. whitmani* and *L. longipalpis* in the peridomicile of houses present in Lençóis National Park. The predominance of *L. whitmani* as well as other potential vectors of *L. braziliensis* could explain the endemicity of TL in the study area. In the same way, the high abundance of *L. longipalpis* could explain the sporadic cases of VL. The study highlights the need for a more effective and permanent surveillance regime in order to control the expansion of vectors of leishmaniasis and to minimize outbreaks of the disease. It is important to stress that it is the health and well-being not only of the inhabitants of the region that is at risk but also of the thousands of tourists that visit the conservation and recreational area of Lençóis Maranhenses National Park. Nature-based tourism provides valuable revenue to sustain local communities and to support conservation, education, and wildlife research; hence it is of utmost importance to prioritize health and safety in this area.

## Abbreviations

TL: Tegumentary leishmaniasis
VL: Visceral leishmaniasis
