# Erratum to: An ecological study of sand flies (Diptera: Psychodidae) in the vicinity of Lençóis Maranhenses National Park, Maranhão, Brazil

**DOI:** 10.1186/s13071-015-1215-5

**Published:** 2015-11-20

**Authors:** Adalberto Alves Pereira Filho, Maria da Conceição Abreu Bandeira, Raquel Silva Fonteles, Jorge Luiz Pinto Moraes, Camila Ragonezi Gomes Lopes, Maria Norma Melo, José Manuel Macário Rebêlo

**Affiliations:** Departamento de Parasitologia, Instituto de Ciências Biológicas, Universidade Federal de Minas Gerais, Belo Horizonte, 31270-901 MG Brazil; Departamento de Biologia, Centro de Ciências Biológicas e da Saúde, Universidade Federal do Maranhão, São Luís, 65080-805 MA Brazil; Departamento de Cartografia, Instituto de Geociências, Universidade Federal de Minas Gerais, Belo Horizonte, 31270-901 MG Brazil

Unfortunately, the original version of this article [1] contained a mistake. In Figure 1, panels C and D were inadvertently included as duplicates of panel B. The correct Figure 1, with the correct panels C and D is included below.

**Fig. 1:**
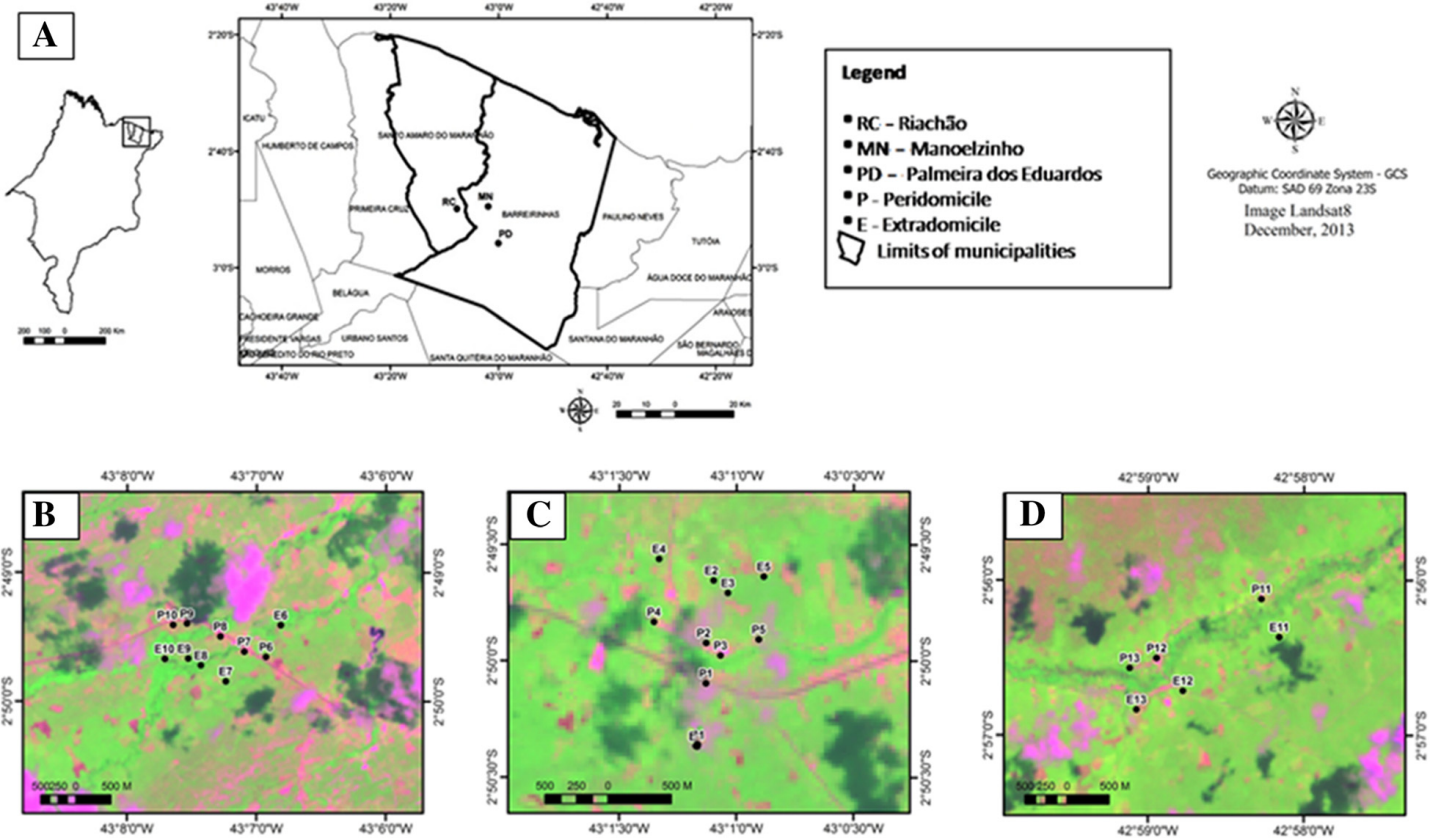
Localization of the Brazilian State of Maranhão and the municipalities in which the Lençóis Maranhenses National Park is situated **a**. Spatial arrangement of the sampling sites located at Riachão in the municipality of Santo Amaro **b**, and at Manoelzinho (**c**) and Palmeira dos Eduardos (**d**) located in the municipality of Barreirinhas
